# Dandruff is associated with the conjoined interactions between host and microorganisms

**DOI:** 10.1038/srep24877

**Published:** 2016-05-12

**Authors:** Zhijue Xu, Zongxiu Wang, Chao Yuan, Xiaoping Liu, Fang Yang, Ting Wang, Junling Wang, Kenji Manabe, Ou Qin, Xuemin Wang, Yan Zhang, Menghui Zhang

**Affiliations:** 1 State Key Laboratory of Microbial Metabolism, Joint International Research Laboratory of Metabolic & Developmental Sciences, School of Life Sciences and Biotechnology; Ministry of Education Key Laboratory of Systems Biomedicine, Shanghai Center for Systems Biomedicine (SCSB), Shanghai Jiao Tong University, Shanghai, 200240, China; 2 Kao (China) Research & Development Center, 623 Zi Ri Road, Minhang, Shanghai, 200241, China; 3 Shanghai Skin Disease Hospital, 1278 Bao De Road, Zhabei, Shanghai, 200443, China; 4 Kao Corporation, Biological Science Laboratories, 2606 Akabane, Ichikai-machi, Haga-gun, Tochigi, 321-3497, Japan

## Abstract

Dandruff is an unpleasant scalp disorder common to human populations. In this study, we systematically investigated the intra- and inter-associations among dandruff, physiological conditions such as sebum of the scalp, host demographics such as gender, age and the region of the scalp, and the microorganisms on the scalp. We found that the physiological conditions were highly relevant to the host age and varied in different regions of the same scalp. The sebum quantity and water content were negatively correlated with the formation of dandruff and had significant relationships with the two dominant but reciprocally inhibited bacteria on the scalp (*Propionibacterium* and *Staphylococcus*). The dominant fungus (*Malassezia* species) displayed contrary roles in its contribution to the healthy scalp micro-environment. Bacteria and fungi didn’t show a close association with each other, but the intramembers were tightly linked. Bacteria had a stronger relationship with the severity of dandruff than fungi. Our results indicated that the severity of dandruff was closely associated with the interactions between the host and microorganisms. This study suggests that adjusting the balance of the bacteria on the scalp, particularly by enhancing *Propionibacterium* and suppressing *Staphylococcus*, might be a potential solution to lessen dandruff.

Dandruff is a common scalp disorder that has occurred for centuries and has a prevalence of nearly 50% in the worldwide population^1^. The formation of dandruff has been studied for decades, but no coincident view has been widely accepted. Dawson and others have proposed that dandruff is the result of individual susceptibility, sebaceous secretion and *Malassezia* fungi^2^,^3^,^4^. Gaitanis has reported that some metabolic products of tryptophan produced by *Malassezia*, such as indole derivatives, cause dandruff^5^. Recently, another study has demonstrated that the disequilibrium in the proportion of the major bacterial and fungal populations are associated with dandruff^6^.

For a long time, studies on dandruff predominantly focused on fungi, particularly the *Malassezia* species, which are major fungi colonizing the human scalp and the dominant members of the cutaneous fungal microbiome^2^,^3^. Of the 14 known cultured species of *Malassezia*, the most clinically significant species are *M. restricta* and *M. globosa*^7^,^8^. These species have been reported to be associated with skin diseases, including dandruff, seborrheic dermatitis, pityriasis dermatitis, and atopic dermatitis^9^. However, another microorganism community composed of bacteria also inhabits the human scalp and includes facultative anaerobic bacteria, such as *P. acnes*, and aerobic bacteria, such as *Staphylococcus*^10^,^11^.

The scalp is covered with pilosebaceous units and sweat glands. Human sebum is a complex mixture of triglycerides, squalene, cholesterol esters, wax esters and cholesterols that are secreted from the scalp^12^,^13^. The secretion of sebum is controlled by sebaceous gland activity, which has been reported to have a strong correlation with scalp flaking disorders^2^. The sebum secretion rate increases throughout a person’s teenage years, reaches the highest in the 15- to 35-year-olds and appears to decline continuously throughout the adult age range^12^,^13^,^14^. Throughout the active period of sebum secretion, the secretion rate is higher in males than in females. However, these host physiological conditions could affect the microbial flora living on the scalp by affecting the scalp microenvironment. Sebum is an important food source for the growth of fungi and bacteria. *Malassezia* produces non-specific lipases that can degrade any available triglycerides^15^,^16^. As a result, the saturated fatty acids in sebum are consumed, and the abundant unsaturated fatty acids are left on the skin. Though recently had been challenged^17^, unsaturated fatty acids had long been considered to penetrate into the stratum corneum and lead to barrier disruption^3^,^18^. The bacterium *P. acnes* can release free fatty acids onto the skin and promote bacterial adherence by hydrolyzing triglycerides and can produce bacteriocins to kill other strains^19^,^20^. A person’s age and gender also contribute to the variability of the microbial flora resident on the skin^10^. Physiological and anatomical differences in the cutaneous environments (i.e., sweat, sebum and hormone production) partially account for the microbial differences between males and females^21^.

Based on the results of previous studies, scalp dandruff could be affected by many factors, including not only the microorganism residents but also many host factors. However, the associations of the severity of dandruff with the composition structure of the microorganisms, host demographics, and host physiological conditions and their interaction have not been clarified to date. In this study, we constructed an association network based on pyrosequencing data of the bacterial and fungal communities on human scalp samples with slight to moderate dandruff (but not severe scaling such as seborrheic dermatitis) to attempt to systemically analyze their relationships.

## Results

### Relationship among dandruff, host demographics and host physiological conditions

The dandruff level was represented with a score from 0–8 on the basis of the adherent scalp flaking scale (ASFS)^22^. According to the dandruff severity, we grouped samples with ASFS scores from 0–2 as the normal group and the rest of the samples as the dandruff group (Supplementary Fig. S1). The host physiological conditions (i.e., sebum, water content and transepidermal water loss (TEWL)) of each sample were also measured. We investigated the effects of host demographics (sampling regions, age and gender) on the degree of dandruff (Table 1) as well as the relationship between dandruff and three physiological conditions (Supplementary Fig. S2). First, dandruff on the top region of the scalp was heavier than dandruff on the side region. The top region also had higher sebum and TEWL but a lower water content. Moreover, dandruff was negatively associated with the sebum (p = 2.21 × 10^−6^) and water content (p = 2.25 × 10^−3^), indicating that the sebum and water content on different regions of the scalp had important relationships with dandruff. The negative relationship between dandruff and sebum might also due to a greater sebum penetration into dandruff scalp stratum corneum^23^. Next, we measured the impact of age on dandruff. Here, the age cutoff value was set at 40, which was somewhat arbitrary but could be informative regarding the secretion of sebum^12^,^13^. We found that the younger group had less dandruff than the older group. Moreover, the younger group had relatively higher sebum than the older group. Considering the negative relationship between dandruff and sebum described above, this result suggested that the secretion of sebum, which is influenced by age, could affect the degree of dandruff. No differences in dandruff, sebum and water content were found between the male and female groups. Although the female group had a higher TEWL, the difference between the two groups was not significant.

We measured the interaction relationships among host demographics on dandruff and physiological conditions. The results indicated that the sampling regions and age had significant interactions with dandruff (p = 8.80 × 10^−3^) and sebum (p = 8.57 × 10^−3^) (Supplementary Fig. S2b,S2c). These relationships suggested that the severity of dandruff represented by the ASFS score was co-affected by many factors instead of a single unique factor.

### Microbiota profiling of the dandruff samples and their interactions

**Figure 1:**
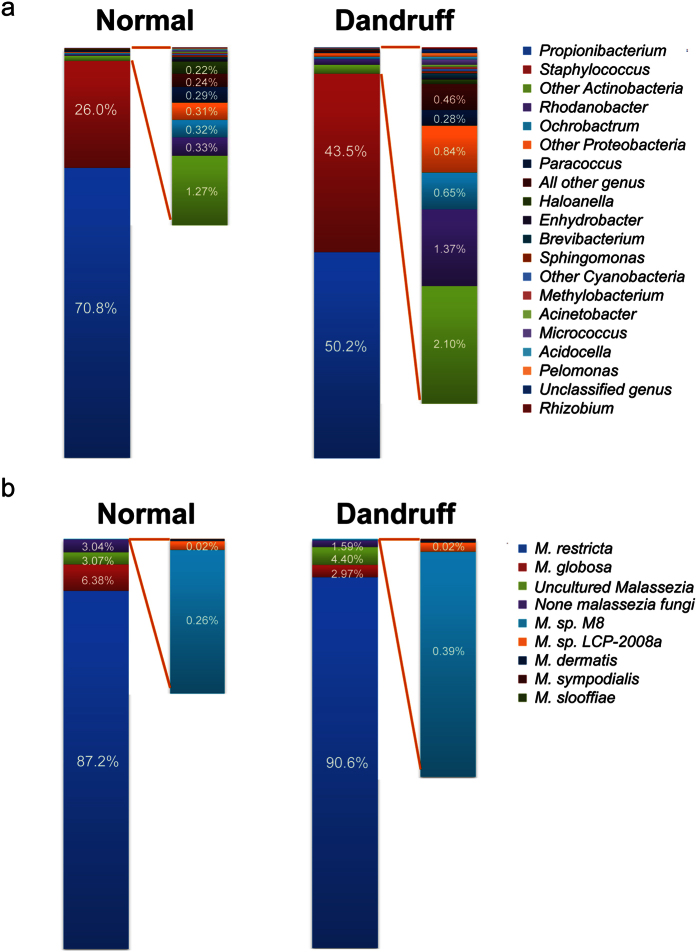
Distribution of the bacterial genera and fungal species in normal and dandruff samples. The distribution of bacteria at the genus level (**a**) and fungi at the species level (**b**) in the normal (left) and dandruff (right) groups. Results are presented as the percentage (%) of total sequences.

Using 454 pyrosequencing, we obtained 1,042,946 high quality reads from the total bacterial 16S rDNA V1–V3 sequences from 170 samples. There was a median of 5,602 reads per sample, with a range from 3,047 to 39,516. A total of 753 operational taxonomic units (OTUs) were identified at a threshold of 97% sequence similarity identity (median = 58 OTUs per sample, ranging from 18 to 193 OTUs). Eleven bacterial phyla were detected, but most sequences were assigned to two bacterial phyla: *Actinobacteria* (64.9%) and *Firmicutes* (32.5%). Of the 123 identified genera, *Propionibacterium* (63.3%, *Actinobacteria*) and *Staphylococcus* (32.4%, *Firmicutes*) comprised more than 95% of the total sequences. A total of 99.7% of the *Propionibacterium* belonged to *P. acne*, and 94.9% of the *Staphylococcus* were *Staphylococcus spp* (including *S. epidermidis, S. capitis and S. caprae*, whose 16S rDNA V1–V3 sequences were highly similar). Interestingly, we found that *Propionibacterium* decreased from 70.8% to 50.2% in the dandruff group (p = 1.89 × 10^−5^), whereas *Staphylococcus* increased from 26.0% to 43.5% (p = 2.12 × 10^−4^) (Fig. 1a); these results were consistent with those of previous studies^6^. Moreover, the proportion of the other low abundance bacteria increased in the dandruff group from 3.2% to 6.4%.

For fungi sequencing, 599,004 high quality ITS reads from 162 samples were obtained, with a median of 3,277 reads per sample and a range from 1,003 to 10,154. A total of 378 OTUs were identified in our study (median = 51 OTUs per sample, ranging from 27 to 102 OTUs). The majority of the fungi on the scalp were *Malassezia*, with 88.5% *M. restricta* and 5.1% *M. globosa*. Several cultured *Malassezia* (*M. sympodialis*, *M. dermatis*, and *M. slooffiae*), uncultured *Malassezia* (*M. sp M8* and *M. sp LCP-2008a*) and other *Malassezia* species accounted for less than 4.0% of the total sequences. The remaining 2.5% were other non-*Malassezia* species. The most predominant fungal species (*M. restricta*) was present on the scalps in both the normal and dandruff groups, with frequencies of 87.2% and 90.6% of the sequences, respectively. *M. globosa*, which followed *M. restricta* in abundance, accounted for 6.4% of the sequences in the normal samples but decreased to 3.0% in the dandruff samples (p = 0.047). The abundance distributions of other *Malassezia* and non-*Malassezia* were quite similar in the two groups (Fig. 1b).

**Figure 2:**
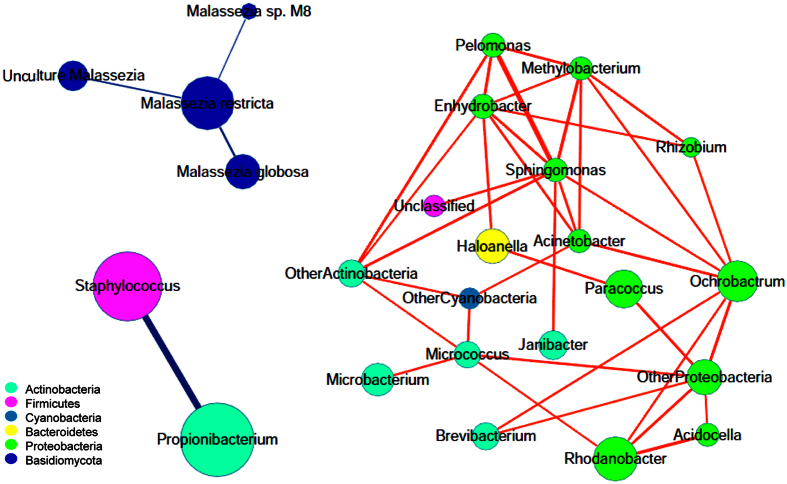
Correlation network of microbiota resident on the scalp. Each node shows one genus of bacteria or one species of fungi. The size of the node corresponds to the log-transformed relative abundance of the microbiota. The thickness of the edges corresponds to the |r| value of the Kendall relationship. The color of the edges corresponds to the positive (red) or negative (blue) relationship. The length of the edges has no meaning.

Next, we used Cytoscape to construct a correlation network of the microbiome resident on the scalp using the Kendall relationship. To obtain the relative robustness, the exhibited relationship threshold was set at |r| > 0.4 and the sample coverage for the shown node was above 20% (Fig. 2). As a result, we obtained three isolated clusters. One cluster was the negative interaction network of *Staphylococcus* and *Propionibacterium*, which were the major commensal bacteria on the scalp. Another cluster consisted of low abundance bacteria that were positively correlated with one another; most of them belonged to two phyla (*Proteobacteria* and *Actinobacteria*) that have been reported to be widely distributed in nature and commonly occur in soil and water^6^. The third cluster was the fungal network, with *M. restricta* as the key member and a negative correlation with other *Malassezia* species. These results indicated that the bacteria and fungi located on the scalp were independent, and each had its own member relationships for competition or mutualistic symbiosis.

### Relationship between the microbiota and dandruff

**Figure 3:**
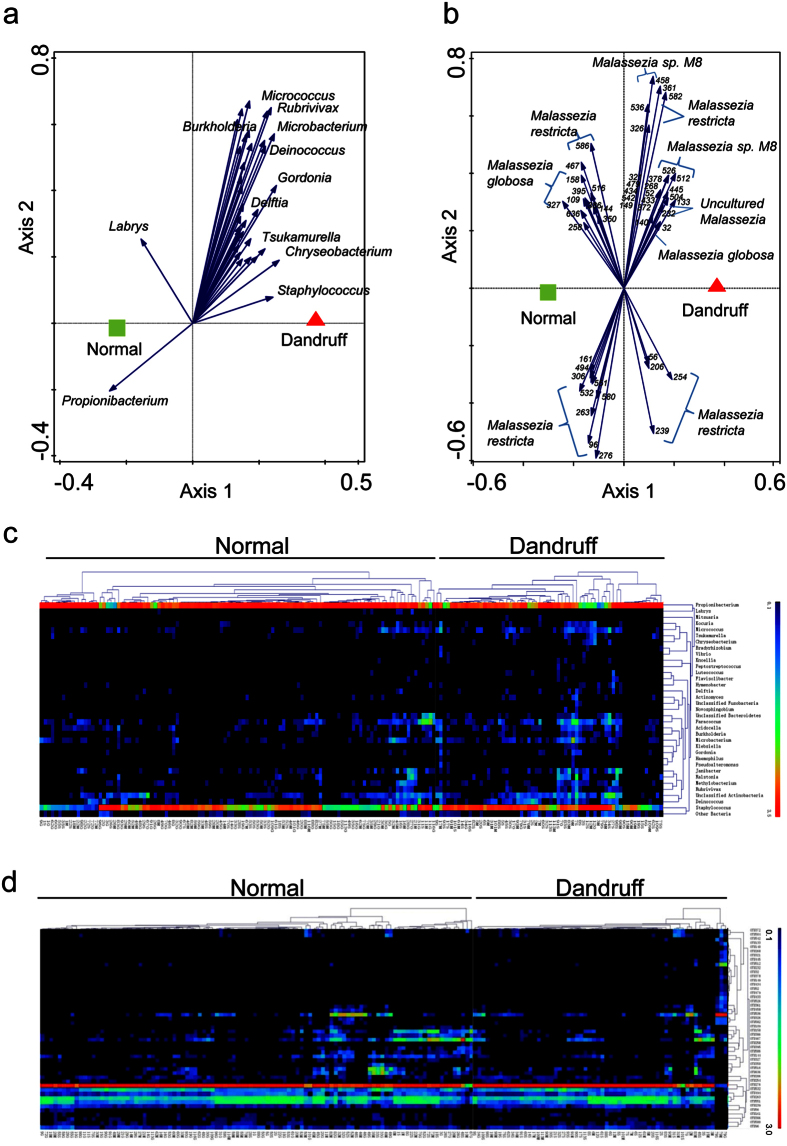
Relationship between the microbiota and dandruff. (**a**,**b**) Redundancy analysis (RDA) of the bacterial genera (**a**) and fungal OTUs (**b**) corresponding to dandruff. Responding genera and OTUs are indicated by blue arrows. The top 10 key bacterial genera and the fungal OTU serial numbers were listed near the arrows. (**c,d**) Heat-map of RDA-identified key bacterial genera (**c**) and fungal OTUs (**d**) corresponding to dandruff. The color of the spot corresponds to the log-transformed relative abundance. The genera or OTUs and samples are clustered according to Kendall correlations.

Redundancy analysis (RDA) identified 35 key bacterial genera (p = 0.002 at a variance contribution of 1.8%) related to the severity of dandruff. Of them, 33 genera including *Staphylococcus* showed a significant positive correlation with dandruff. In contrast, only two genera (*Propionibacterium* and *Labrys*) showed a significant negative correlation with dandruff (Fig. 3a,c). Because *Staphylococcus* and *Propionibacterium* were the two dominant but reciprocally inhibited bacterial genera on the scalp, these results indicated that dandruff was mainly associated with the balance of these two genera.

For the fungi, we found that the relationship between the fungal community at the species level and dandruff was very weak (p = 0.272 by RDA). The relationships obtained at the OTU level tended to be stronger but were still not significant (p = 0.06). A total of 27 OTUs showed a significant positive correlation with danduff (by RDA at the variance contribution of 0.8%). These 27 OTUs belonged to *M. restricta* (n = 11), *M. sp. M8* (n = 7), *M. globosa* (n = 1) and uncultured *Malassezia* (n = 8). Another 21 OTUs were significantly negatively correlated with dandruff; these OTUs belonged to *M. globosa* (n = 7) and *M. restricta* (n = 14) (Fig. 3b,d). Notably, the relationship between the OTUs of *M. restricta* and dandruff showed some inconformity. Fourteen OTUs of *M. restricta* were negatively correlated with dandruff, including OTU276 (the most abundant OTU, which belongs to *M. restricta isolate HA414*, GenBank EU00587.1) and OTU263 (the second most abundant OTU, which belongs to *M. restricta isolate ITC58*, GenBank KJ412033.1). An additional 11 OTUs showed a positive relationship with dandruff, including OTU536 (the third most abundant OTU, which belongs to *M. restricta isolate POL.1.11.II*, GenBank KC152885.1). These results suggested that different strains of *M. restricta* might have opposing contributions to dandruff, and analysis at the strain level might be more precise for studies of the relationship between fungi and dandruff.

### Relationship between the microbiota and host factors

Redundancy analysis was performed on the relationship between host factors (demographics and physiological conditions) and bacteria (Table 2 and Supplementary Table S1). This analysis indicated that the bacteiral community was significantly affected by the age, gender, sebum and TEWL and showed weak relationships with the sampling region and water content. Of the two major genera, *Propionibacterium* was affected by the three host demographics, sebum and water content and prefered to exist on the side region, in males and the younger group. It also showed positive relationships with the sebum and water content. *Staphylococcus* was affected by the sampling region, TEWL and water content, showed a higher ratio with the top region of the scalp, and was positively associated with the TEWL and negatively associated with the water content.

However, only the gender of the host showed a significant correlation with the fungal community (p = 0.03, at the contribution of 0.9%). A total of 16 OTUs were positively correlated with males, and 7 OTUs showed positive relationships with females. The other host factors had no significant relationships with the fungal community (p > 0.05) (Table 2 and Supplementary Table S2).

## Discussion

**Figure 4:**
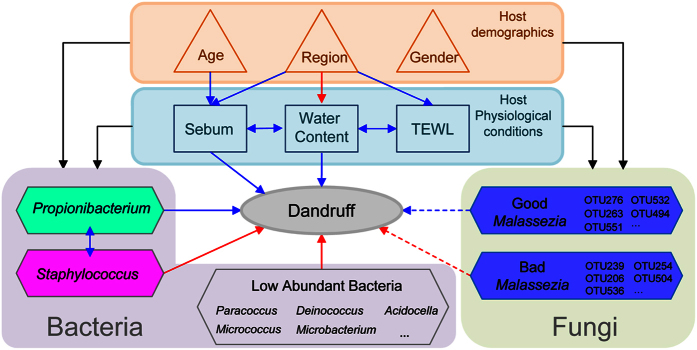
Overview of the relationships among dandruff, host demographics, physiological conditions and microorganisms. The shape of the nodes corresponds to the type of each factor. The color of the edges corresponds to the positive (red), negative (blue) or sole effect (black) relationship. The shape of edges corresponds to the interaction (↔) or effect (→) of the relationship. The full line of the edges corresponds to P < 0.05, and the imaginary line corresponds to P > 0.05.

In this study, we provide the first systematic view of the relationship among the host physiological conditions, demographics, commensal microbiota and dandruff. We found that dandruff was associated with the interactions between the host and the microorganisms on the human scalp (Fig. 4).

Microorganisms on the scalp, especially fungi, have been predominantly thought to be the main cause of the development of dandruff. In this work, we were surprised to observe that there was not a close association between the bacteria in genus and fungi in species. Furthermore, the relationship between bacteria and dandruff was stronger than the relationship between fungi and dandruff. The most abundant bacteria on the scalp (*Propionibacterium* and *Staphylococcus*) showed reciprocal inhibition with each other, concurrent with Clavaud and Wang’s works^6^,^24^. This finding may be explained because *Propionibacterium* can secrete bacteriocins to suppress the growth of *Staphylococcus*^25^, whereas *Staphylococcus* can mediate the fermentation of glycerol and inhibit the overgrowth of *Propionibacterium*^26^. Compared with a normal scalp, the dandruff region had decreased *Propionibacterium* and increased *Staphylococcus*, suggesting that the balance between *Propionibacterium* and *Staphylococcus* might be important to the severity of dandruff. Clavaud has recently noted that the disequilibrium in the proportion of the major bacterial and fungal populations was associated with dandruff^6^. Our result is somewhat different in that we did not find close linkages between the bacteria and fungi. Moreover, overall fungi did not exhibit an important role in the severity of dandruff at either the species or OTU level. This discordance in the evaluation may be partially due to the functional diversity within the fungal species. Indeed, we found that different OTUs of the same *Malasseiza* species showed opposing relationships with dandruff, which consists with the studies in Brazilian and Japanese population that different *Malassezia* subtypes were found in different proportions in samples^27^,^28^. This finding suggests that not all of the *Malassezia* are bad for healthy scalps. Thus, studies of the relationship between fungi and dandruff might be more reasonable at the OTU level instead of the species level.

Our study revealed that the physiological conditions were highly relevant to the host age and varied in different regions of the same scalp. Scalp sebum could act as a food source for *Propionibacterium*, and a high water content provided a suitable environment for *Propionibacterium* growth. The relationship between the severity of dandruff and the bacteria suggested that adjusting the equilibrium of the bacteria, particularly by increasing the *Propionibacterium* and decreasing the *Staphylococcus* on the scalp, might be a novel way to lessen the severity of dandruff. The interactions among host factors and microorganisms imply that regulating host physiological conditions may also be a solution to inhibit the development of dandruff.

## Materials and Methods

### Volunteer recruitment

Volunteers with overall healthy physical condition but varying dandruff levels were recruited from the Shanghai Skin Disease Hospital (Shanghai, China), which holds a big biobank collecting special samples for scalp and hair test. The bank had been more than two years before our study and all subjects should not use any anti-dandruff products once they joined in the bank. In our survey, 363 subjects in total participated the prescreening process with the inclusion criteria: (1) healthy female or male aged between 18 and 60 years; (2) provided written informed consent willing to participate in complete test process; (3) no hair perming or coloring in the last two months and promised no hair perming or coloring during the study period; (4) the last shampoo was performed 48 ± 2 hours before the formal test. Subjects were excluded if they (1) joined other clinical research in last 3 months; (2) during pregnant or lactation period; (3) had pregnant willing during test period; (4) Body Mass Index (BMI) > 30; (5) suffered from seborrheic dermatitis, psoriasis, allergic dermatitis or eczema; (6) had scar, inflammation, tattoo or other diseases that may interfere with the results of our study; (7) accepted anti-tumor, immunosuppressant or radiation therapy; (8) used external drug or hormone on scalp in last 3 months. After screening, 60 qualified subjects joined the formal test and 59 subjects finished the test. Their physiological information was recorded (Table 1).

The study was approved by the Scientific and Ethical Committee at the Shanghai Skin Disease Hospital. All experiments were performed in accordance with the approved guidelines and regulations. All of the volunteers signed the informed consent, which explained the procedure and purpose of the study.

### Sample collection

#### Sample Region & ASFS Score Determination

All of the subjects washed their hair 48 hours prior to sample collection and did not use any anti-dandruff products. The scalp of each subject was divided into eight divisions by a licensed dermatologist^22^,^29^, with four divisions of the top scalp defined as “top” and the other divisions defined as “side”. The dandruff level of each division was graded according to the grading approach of the adherent scalp flaking scale (ASFS) (Supplementary Fig. S1)^22^. For each subject, 3 divisions with minimum, medium and maximum ASFS scores were marked for sampling.

#### Host Physiological Measurements

The physiological measurements were performed under a controlled environment with relative humidity between 40–60% and temperature at 25 °C. All subjects stayed at this environment for 30 minutes before the measurements. The sebum of each marked area was examined with a Sebumeter SM815 (Courage+Khazaka Electronic GmbH, Cologne, Germany) using a square probe 8 mm in length and 8 mm in width. In brief, to avoid interference of hair at the each measuring site, the hair was parted by comb and secured with hair clips and then the tape on probe was pressed slightly onto the scalp for 30 seconds to measure sebum. The water content (W value) and barrier function (bP value) of each marked area were examined five times with the ASA-MX (Asahi Techno Lab)^30^, a patented instrument based on low and high frequency alternating voltages applied to the skin surface, using a probe 2 mm in diameter, and the mean values were used for statistical analysis (Table 1). In brief, subjects held electrode with hands and then the probe was pressed slightly onto the scalp for several seconds until values displayed on the panel. A significant correlation (r = 0.53, p < 0.01) was found between the bP value and TEWL determined by the VapoMeter (Delfin Technologies Ltd., Kuopio, Finland). There was also a significant correlation (r = 0.54, p < 0.01) between the W value and water content measured by the Corneometer (Courage+Khazaka Electronics, Cologne, Germany).

#### Sampling

The sample collection was performed on the previously marked division of each subject using swabs based on the procedure proposed by Gemmer *et al.*^31^ with minor modifications. The swabs were frozen immediately after sampling, stored at −80 °C and then used for bacterial and fungal DNA extraction.

### Extraction of bacterial and fungal DNA from swabs

Bacterial and fungal DNA were extracted using the DNeasy Tissue kit (Qiagen, 69506) according to the provided protocols with minor modifications^32^. The DNA content was measured by the Qubit dsDNA HS Assay kit (Invitrogen, Q32851).

### Pyrosequencing

The barcoded pyrosequencing was performed with the Roche 454 FLX+ automated pyrosequencer by Personal Biotechnology Co., Ltd. (Shanghai, China). The bacterial 16S rRNAV1–V3 region (~470 nucleotides) was amplified with the primers 27-F (5′-AGAGTTTGATCCTGGCTCAG-3′) and 533-R (5′-TTACCGCGGCTGCTGGCAC-3′)^33^. The fungal ITS region (~750 nucleotides) was amplified with the primers ITS1-F (5′-CTTGGTCATTTAGAGGAAGTAA-3′) and ITS4-R (5′-TCCTCCGCTTATTGATATGC-3′)^34^. The obtained 454 sequences were archived at the NCBI Sequence Read Archive (SRA) under accession number SRP057641.

### Bioinformatics and statistical analysis

Sequence processing was performed using QIIME 1.7.0^35^ with the default parameters. Briefly, sequences were quality trimmed, and chimeric sequences were identified with ChimeraSlayer and removed. Then, the sequences were clustered into operational taxonomic units (OTUs) using the program CD-HIT with a minimum identity of 97%^36^. Samples were rarefied at 3047 and 1003 sequences per sample for the bacterial 16S rRNA and fungal ITS, respectively. OTUs with at least 3 reads for 16S and 1 read for ITS were kept for further analysis.

The representative sequences of the 16S rRNA genes from each OTU were aligned using PyNAST^31^ and the Greengenes database^37^. Taxonomy was assigned using the Ribosomal Database Project (RDP) classifier at an 80% confidence level^38^. The representative sequences of the fungal ITS genes from each OTU were aligned using DNASIS Taxon (Hitachi Software Engineering Co. Ltd., Tokyo, Japan) and the GenBank data offered by NCBI (http://www.ncbi.nlm.nih.gov/).

Redundancy analysis (RDA) was performed via Canoco for Windows 5 (Microcomputer Power, NY, USA)^39^. Permutational multivariate analysis of variance (PERMANOVA) was performed via R-vegan3.0.2 (http://vegan.r-forge.r-project.org/). The correlation analysis and one-way analysis of variance (ANOVA) were performed in MatLab^®^ (2010b, The MathWorks, Natick, MA, USA) environment. Network drawing was performed in Cytoscape 3.1.1 (http://cytoscape.org/).

## Additional Information

**How to cite this article**: Xu, Z. *et al.* Dandruff is associated with the conjoined interactions between host and microorganisms. *Sci. Rep.* **6**, 24877; doi: 10.1038/srep24877 (2016).

## Supplementary Material

Supplementary Information

Supplementary Table S1

Supplementary Table S2

## Figures and Tables

**Table 1:** Sample information and physiological characteristics of recruited volunteers.

		ASFS score		Sebum (μg/cm^2^)		Water Content		TEWL	
Host demographics		Median/Range	*P*-value^a^	Median/Range	*P*-value^a^	Median/Range	*P*-value^a^	Median/Range	*P*-value^a^
Region	Top(n = 98)	2/0-8	0.0389	98/1–273	0.0161	3.22/1.16–8.52	2.70 × 10^−3^	71.25/8.04–723.63	1.33 × 10^−6^
	Side(n = 76)	1/0–8		83/15–169		4.90/1.10–22.70		23.55/7.63–634.16	
Age (year)	20–39(n = 96)	1/0–8	0.0308	98/14–273	2.84 × 10^−4^	3.75/1.10–13.25	0.324	51.27/7.83–667.80	0.286
	40–59(n = 78)	2/0–8		70/3–214		3.81/1.16–22.70		45.88/7.36–723.63	
Gender	Female(n = 115)	2/0–8	0.332	94/1–273	0.502	3.78/1.10–22.70	0.501	61.34/7.36–723.63	0.294
	Male(n = 59)	2/0–8		90/3–221		3.75/1.16–13.52		35.72/8.10–634.16	

^a^The *P*-value by ANOVAN was corrected via the false discovery rate (FDR).

**Table 2:** Relationship between microbiota and host factors.

Host factors	Bacteria			Fungi		
	*P*-value^a^	Positive^b^	Negative^b^	*P*-value^a^	Positive^b^	Negative^b^
Region^c^	0.104	4	31	0.058	13	16
Age	0.002	29	2	0.202	15	9
Gender^c^	0.006	20	1	0.030	16	7
Sebum	0.002	1	27	0.194	15	9
Water content	0.150	20	9	0.162	14	41
TEWL	0.036	32	0	0.782	18	12

^a^The *P*-value was calculated by redundancy analysis (RDA).

^b^The number of the bacterial genera or fungal OTUs positively or negatively associated with the physiological characteristics.

^c^The sampling region was calculated from top to side, and the gender was from male to female.
