# Supplementary material for: Dandruff is associated with the conjoined interactions between host and microorganisms
Source: Sci Rep. 2016 May 12;6:24877. doi: 10.1038/srep24877 (PMC4864613; doi:10.1038/srep24877)

**Supplementary Information**

# Dandruff is associated with the conjoined interactions between host and microorganisms

Zhijue Xu[(1)#], Zongxiu Wang[(2)#], Xiaoping Liu[(3)#], Fang Yang[(1)], Ting Wang[(2)], Junling Wang[(2)], Kenji Manabe[(4)], Chao Yuan[(3)], Ou Qin[(3)], Xuemin Wang[(3)*], Yan Zhang[(1)*], Menghui Zhang[(1)*]

[(1)] State Key Laboratory of Microbial Metabolism, Joint International Research Laboratory of Metabolic & Developmental Sciences, School of Life Sciences and Biotechnology; Ministry of Education Key Laboratory of Systems Biomedicine, Shanghai Center for Systems Biomedicine (SCSB), Shanghai Jiao Tong University, Shanghai, 200240, China

[(2)] Kao (China) Research & Development Center, 623 Zi Ri Road, Minhang, Shanghai, 200241, China

[(3)] Shanghai Skin Disease Hospital, 1278 Bao De Road, Zhabei, Shanghai, 200443, China

[(4)] Kao Corporation, Biological Science Laboratories, 2606 Akabane, Ichikai-machi, Haga-gun, Tochigi, 321-3497, Japan

[#] These authors contributed equally to this work.

[*] Correspondence should be addressed to Prof. Menghui Zhang (E-mail: mhzhang@sjtu.edu.cn); Prof. Yan Zhang (E-mail: yanzhang2006@sjtu.edu.cn); and Prof. Xuemin Wang (E-mail: dermxmwang@163.com)

**Figure legends**

Supplementary Fig. S1 Representative images of ASFS scores of 0, 2, 4, 6 and 8.

Supplementary Fig. S2 Relationships among ASFS Scores and host factors. (a-d) Box plot of the interaction of host demographics on the degree of dandruff and three physiological conditions. The color of the boxes corresponds to the gender of each subgroup sample (red represents female and blue represents male). (e) The correlation of the physiological conditions and ASFS score via RDA.

Supplementary Fig. S1

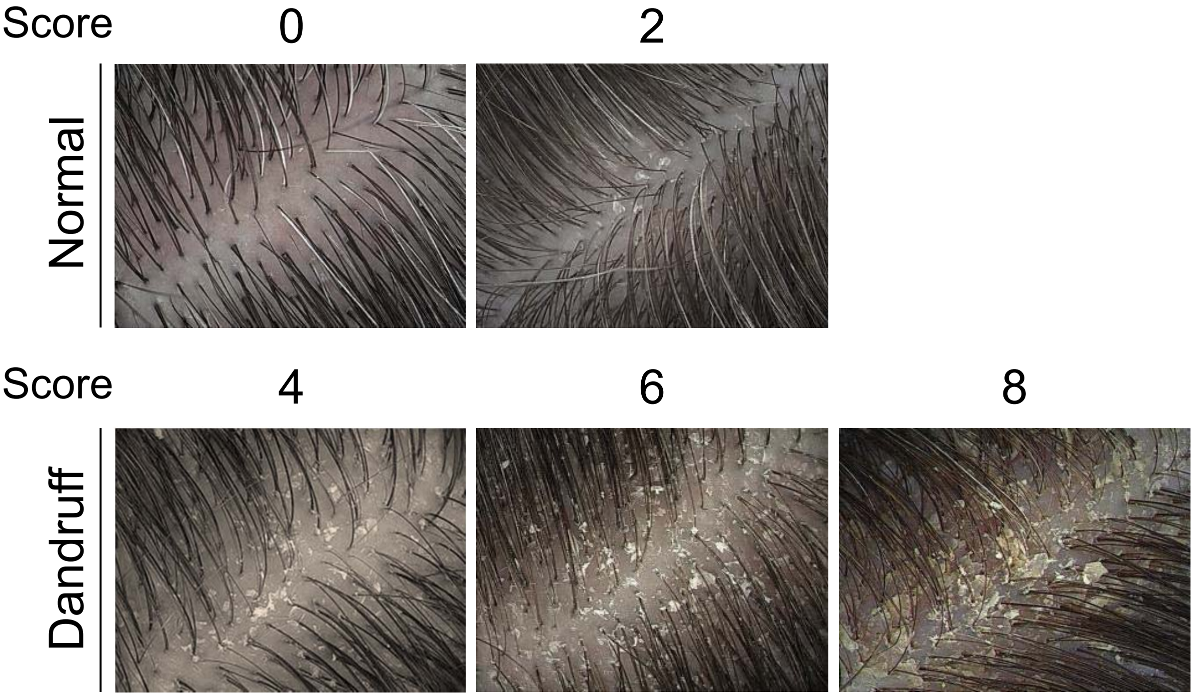

# Supplementary Fig. S2

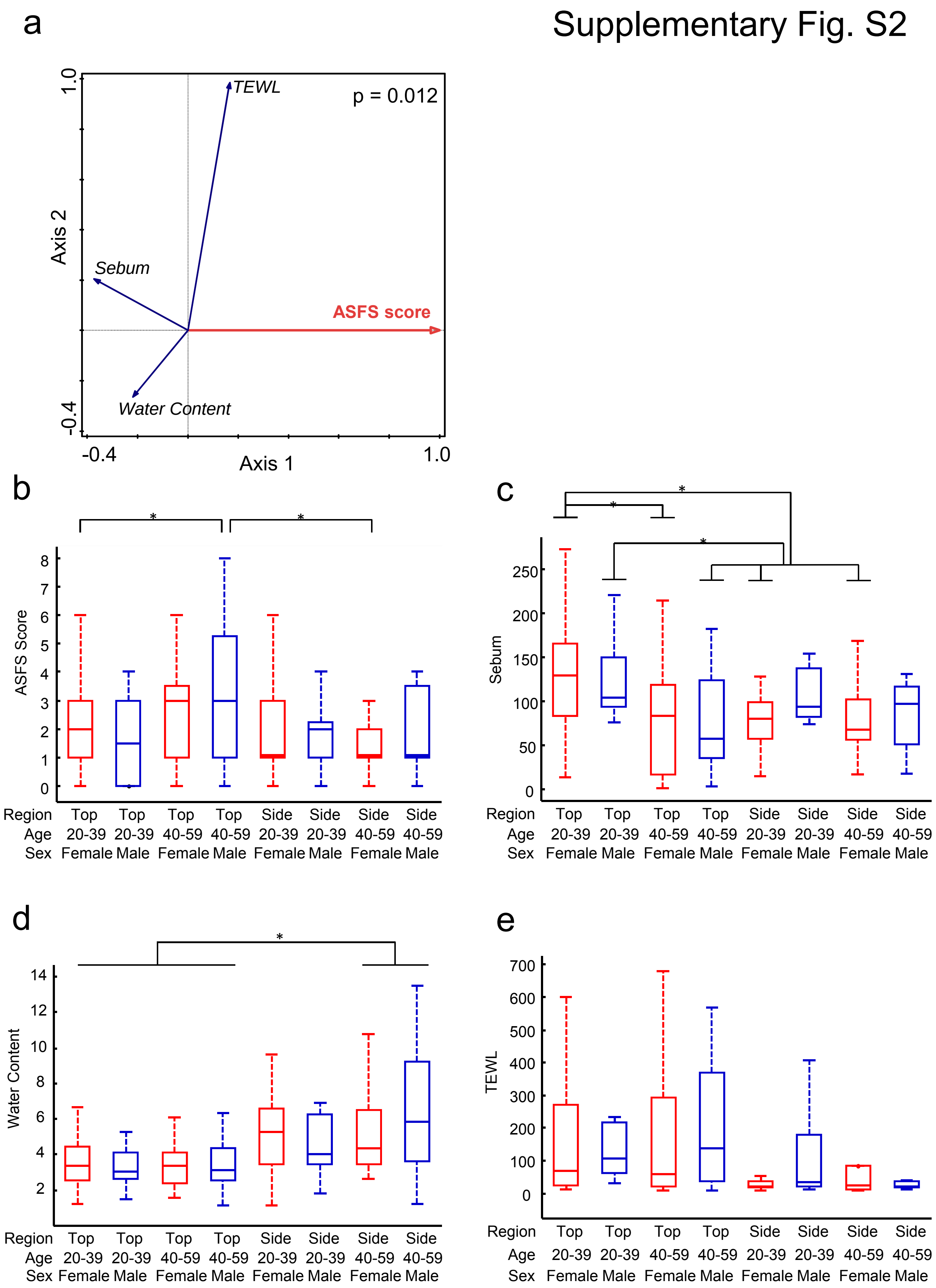

Supplement: Supplementary Information [file srep24877-s1.pdf]
